# Selection constrains lottery assembly in the microbiomes of closely related diatom species

**DOI:** 10.1038/s43705-022-00091-x

**Published:** 2022-02-01

**Authors:** Willem Stock, Anne Willems, Sven Mangelinckx, Wim Vyverman, Koen Sabbe

**Affiliations:** 1 Laboratory of Protistology and Aquatic Ecology, Department of Biology, Ghent University, Krijgslaan 281 – S8, B-9000 Ghent, Belgium; 2 Laboratory of Microbiology, Department of Biochemistry and Microbiology, Ghent University, K.L. Ledeganckstraat 35, B-9000 Ghent, Belgium; 3 SynBioC, Department of Green Chemistry and Technology, Faculty of Bioscience Engineering, Ghent University, Coupure Links 653, B-9000 Ghent, Belgium; 4 Present Address: Phycology Research Group, Department of Biology, Ghent University, Krijgslaan 281 – S8, B-9000 Ghent, Belgium

**Keywords:** Microbial ecology, Microbial communities, Environmental microbiology

## Abstract

It is generally recognised that interactions between microalgae and bacteria play an important role in the functioning of marine ecosystems. In this context, increasing attention is paid to the processes that shape microalga-associated microbiomes. In recent years, conflicting evidence has been reported with respect to the relative importance of selective vs neutral processes in the assembly process. Whereas some studies report strong selection imposed by the host, others propose a more neutral, lottery-like assembly model according to which the chance of bacteria becoming part of the microbiome is proportional to their abundance in the environment and not driven by the selectional pressure created by the host. In the present study, we investigated to what degree selective vs neutral assembly processes constrain taxonomic, phylogenetic and functional variation within and between microbiomes associated with 69 isolates belonging to the *Cylindrotheca closterium* benthic marine diatom complex. The diatom cultures were initiated from non-axenic clonal isolates from different marine environments and geographic locations, and were then reared in a common garden (lab) environment. An important environmental imprint, likely due to in situ lottery dynamics, was apparent in the diatom microbiomes. However, microbiome assembly was also phylogenetically and functionally constrained through selective filtering related to the host microhabitat. Randomised microbiome assembly simulations revealed evidence for phylogenetic overdispersion in the observed microbiomes, reflecting an important role in the assembly process for competition between bacteria on the one hand and predominantly genetically driven differences between the hosts on the other hand. Our study thus shows that even between closely related diatom strains, host selection affects microbiome assembly, superimposing the predominantly stochastically driven recruitment process.

## Introduction

Diatoms are an abundant and highly diverse group of eukaryotic microalgae [1]. Close interactions with bacteria are likely one of the reasons behind their evolutionary success [2]. These interactions also have major ecological implications as they affect food web structure and mediate biogeochemical cycling in both planktonic and benthic marine systems [3–6]. Marine diatom and bacterial community structure, while being spatially and temporally highly variable, appear to be closely linked [7–9]. Changes in interacting partners are likely to have profound effects on the marine nutrient fluxes due to the highly specific nature of these interactions [10–14]. A good understanding of the processes governing the structure of diatom-bacteria associations is therefore important.

Both deterministic and stochastic processes determine which bacteria are associated with a diatom [15, 16, 17]. In the absence of clear deterministic processes, the chance of particular bacteria establishing is proportional to their colonisation rate (i.e. a lottery dynamic; [16]). Although the lottery colonisation dynamic is explicitly stochastic, it can coincide with deterministic processes such as competition, facilitation and other bacterial-bacterial interactions that may skew the odds of bacterial establishment [18]. The functional makeup of the community might be constrained by the number of niches available [19] and diatoms might steer the colonisation process [20] by producing secondary metabolites [21], further shifting the balance from stochastic towards deterministic processes. The cooccurrence of multiple processes simultaneously acting on the assembly process make it challenging to distinguish deterministic and stochastic processes [22]. Diatoms have often been reported to be associated with a species-specific bacterial community [6, 23–26]. Other studies, however, have disclosed high variability between microbiomes of isolates belonging to the same diatom species [27–29]. The contrast between studies reporting species-specific, reproducible bacterial communities [30] and those reporting a large variability in the communities associated with a single species might in part be due to differences in their study design [16, 24, 27] which can impact the assembly process. Studies reporting species-specific and often reproducible diatom-associated microbiomes generally use a common garden approach in which diatom isolates, after having been cured of bacteria, are reseeded with a bacterial inoculum from which bacteria are recruited. Studies reporting high intraspecific variability in microbiome composition usually analyse in situ assembled bacterial communities of diatoms (e.g. [27, 31]). The axenization based approach, may overestimate the role of species-specific selection by eliminating the effect of ecological processes that affect diatom microbiomes in dynamic and complex natural settings. Studies based on non-axenized diatom isolates may fail to recognise selection effects as these can be masked or altered by strong (in situ) environmental imprints on the microbiomes [32, 33].

**Fig. 1:**
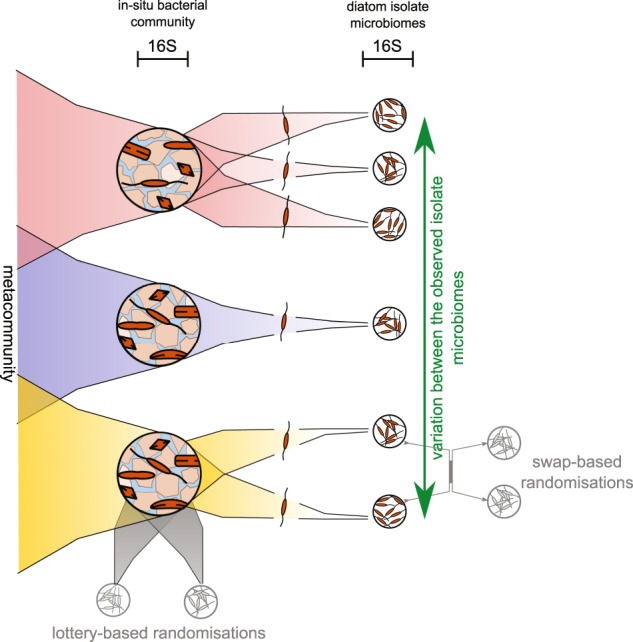
Study design. The microbiome communities of the *C. closterium* isolates and the bacterial communities from the original in situ samples (from which the isolates were originally isolated) were characterised using 16 S rRNA gene amplicon sequencing. The different colours represent different locations (note that different numbers of isolates were obtained from these different localities). Variability in the observed diatom microbiomes is compared to that in randomised communities (grey). Swap-based randomisations (between communities of isolates derived from the same sampling locality) allow evaluating the null hypothesis of no differential host selection effect; the lottery-based randomisation allow evaluating deviations from stochasticity (see text).

In this study we focus on the bacterial microbiome associated with the marine diatom *Cylindrotheca closterium* (Ehrenberg) Reimann & Lewin. Recently, it was demonstrated that *C. closterium* is genetically diverse and comprises several cryptic species [34–36]. Members of this complex are frequently abundant in coastal regions worldwide, both in the water column and in sediments [37, 38]. Our aim was to identify the role of diatom driven selection in minimising variation on the assembly of the diatom associated bacterial communities (Fig. 1). By rearing in situ collected *C. closterium* cells in a common garden environment (controlled laboratory setting) for two consecutive growth cycles prior to characterising their bacterial communities, we aimed at capturing both the imprint of microbiome-shaping processes occurring in the in situ environment of the isolates, and the effect of strain-specific selection processes which could especially become apparent in the common garden setting. In situ and strain-associated bacterial communities were characterised by high-throughput 16S rRNA gene amplicon sequencing. We identified the role of the host selection in the microbiome assembly process by comparing observed to randomised microbiome communities. The latter were obtained [1] by randomising between the communities (‘swap procedure’) of the different diatom isolates, nullifying any differences in the communities related to differential selection by the host isolates, and [2] by de novo assembling randomised communities from the in situ bacterial communities in order to mimic a neutral, lottery-based assembly mechanism (‘lottery procedure’).

Diatom microbiomes are generally dominated by a few bacterial groups (Rhodobacterales, Alteromonadales and Bacteroidetes) but the species representing these groups and their relative abundances are highly variable within and between studies [3] (e.g. 27, 29, 30). We hypothesised that variability between diatom-associated microbiomes would reflect both stochastic and host-related selection processes but that the latter should be most pronounced due to the two consecutive growth cycles under a common garden setting. As a result, we expected the communities coming from different environments to be more similar than the de novo lottery-based randomisations that strictly reflect the differences between environments. Since all hosts are phylogenetically closely related and belong to the same species complex, we expected differences in microbiome structure between the hosts to be small but significant, in line with previous studies that reported such differences, even between diatoms from the same genus [13, 26]. We therefore hypothesised that variation observed between the communities would be slightly larger than the variation between the randomised communities obtained from the swapping procedure.

## Material and methods

### Sampling and initial sample processing

**Fig. 2:**
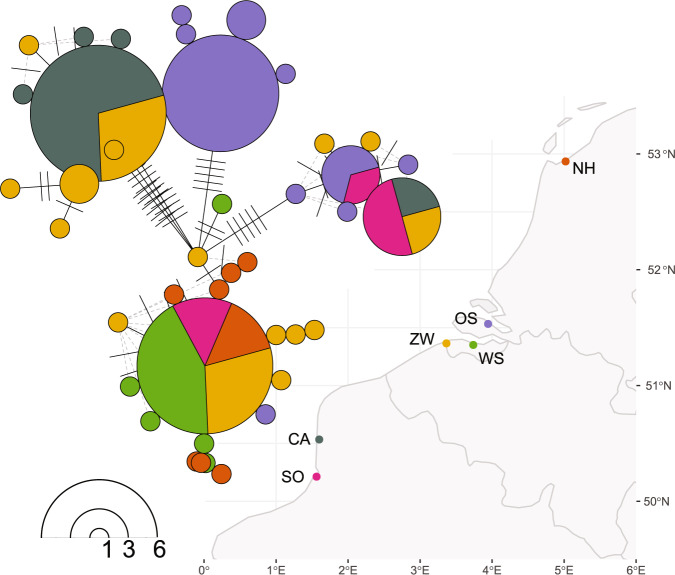
*Cylindrotheca closterium* haplotypes in relation to the location they were isolated from. Bubble size in the network is proportional to the number of isolates showing that haplotype (bottom left). The number of differences between ITS sequences is indicated by dashes. The locations (supplementary table 1) are indicated by different colours.

Sediment and water samples were taken between May and July 2014 from six different estuarine locations in the North Sea and the English Channel (Supplementary Table 1; Fig. 2). Sediment samples were taken with a 6.4 cm diameter Plexiglas core. The centre (3 cm diameter) of the top centimetre of the core was subsampled and stored on ice until processing in the lab. The remaining sediment from the top centimetre, designated for nutrient analysis, grain size analysis and total organic matter content (%TOM) determination, was immediately frozen until processing. Water samples of the incoming tide were collected and stored on ice. Salinity of the interstitial water and flood tide water was measured in situ with a hand-held refractometer (Atago, Japan).

The sediment samples (core centres, cf. above) were thoroughly homogenised upon arrival in the lab. Half of the sediment was stored at −80 °C for bacterial community analysis. The other half was used to isolate diatoms using the lens tissue method [39]. For this purpose, the sediment was spread out in a Petri dish (60 × 15 mm Cellstar^®^ Greiner Bio-one, Austria) and covered with two layers of sterile lens tissue (Whatman) and a sterile coverslip (20 × 40 mm, VWR). After an incubation period of 24 h, at 18 °C with a 12/12 light cycle (20–25 µmol photons/m^2^/s), the coverslip was removed and gently rinsed with autoclaved North Sea water (NSW; 33PSU). The attached epipelic diatoms were collected in a Petri dish filled with NSW and allowed to settle for several hours until single cell isolation.

Upon arrival in the lab, the water samples were homogenised and subsamples for bacterial community composition and nutrient analysis were stored at −80 °C and −20 °C, respectively. The rest of the sample was used to isolate diatoms. The diatoms were allowed to settle overnight at 4 °C, in the dark. The next day, some of the precipitate was collected and diluted in a Petri dish filled with autoclaved NSW. The diatoms were allowed to settle for several hours until single cell isolation.

### Environmental variables

The depth of the oxic layer was measured in the field based on sediment colour of the core immediately after sampling. Grain size analysis (median grain size) was done with a Coulter Counter LS Particle Size Analyser (Beckman Coulter, IN-USA). Concentrations of ammonium, silicate, nitrate, nitrite and phosphate in the (interstitial) water were measured with an automatic chain (SAN plus Segmented Flow Analyser, SKALAR, the Netherlands) after filtration of the samples on Whatman GF/F filters. Percentage total organic matter (%TOM) was measured by calculating the weight loss after combustion (550 °C for 2 h) of the hot air (60 °C) dried sediment.

### Monoclonal diatom cultures

Single *Cylindrotheca closterium* cells were isolated from the Petri dishes by micropipetting [40]. Briefly, single cells were resuspended with a clean needle and brought to the surface from where they were pipetted into a well (96 well plate Cellstar^®^ Greiner) filled with NSW supplemented with F/2 (Sigma-Aldrich, Germany). The well plates were placed at 18 °C, with a 12/12 light cycle (20–25 µmol photons/m^2^/s of cool fluorescent white light) and inspected regularly. Cultures in which contaminants (e.g. algae other than the single isolated diatom cell) were observed were discarded. Several additional pennate diatoms, other than *C. closterium*, were isolated in a similar fashion to serve as controls (Supplementary table 1). Once the monoclonal cultures were dense, i.e. the cells covered most of the well, the medium was refreshed prior to transfer of the culture to a new well (12 well plate Cellstar^®^ Greiner). After 3–4 days, the medium of late exponential cultures was refreshed again before they were harvested. One millilitre of resuspended culture was used for DNA extraction and another millilitre was fixed (final concentration of 4% formaldehyde) for flow cytometer analyses.

### DNA analysis and phylogenetic relatedness of the diatom isolates

DNA of the diatom cultures and environmental samples was extracted using the phenol-chloroform method as in Muyzer et al. [41]. For the bacterial community analyses, the V1-V3 hypervariable regions of the 16S rRNA gene were amplified using pA (AGAGTTTGATCCTGGCTCAG, positions 8-27) and BKL1 (GTATTACCGCGGCTGCTGGCA, positions 536-516) primers as in Tytgat et al. [42]. PCR and library prep were done according to D’hondt et al. [43]. Sequencing was done on an Illumina MiSeq system (300 bp paired-end). Artificial mock communities, blanks and duplicate samples were included for quality control. The obtained forward and reverse reads of 16S rRNA gene sequences were merged using the software program Paired-End Read Merger (PEAR version 0.9.4; [44]). The merged reads were further processed in Usearch8 [45]. Clustering was done on the dereplicated reads with the singletons removed. Chimeras were detected de novo and by mapping to the GOLD database and removed. An Operational Taxonomic Units (OTU) table was then constructed using a 3% cut-off. OTUs were classified with IDTAXA [46], implemented in the R package DECIPHER (version 2.12.0; [47]), using the Silva 138.1 reference database [48]. The OTU sequences were aligned with SSU-ALIGN (version 0.1.1; [48]), simultaneously masking ambiguously aligned base positions. The alignment was used to construct a phylogenetic tree of the OTUs using IQ-TREE (version 1.6.10;[49, 50, 51]). This tree was further used to estimate the phylogenetic distances between OTUs (see below).

The phylogenetic relatedness between the collected *Cylindrotheca* strains was inferred from two genetic markers. The nuclear ITS region (ITS1-5.8S-ITS2) was obtained for every strain according to Vanelslander et al. [52]. The chloroplast (chl) 16 S rRNA gene sequences were acquired for the 16S rRNA gene MiSeq dataset: the most abundant sequence in the dereplicated read files was extracted and manually checked for its resemblance to known chloroplast sequences. Each marker (ITS and chl 16 S) was aligned by ClustalW using MEGA 7. The alignments were manually curated and afterwards joined using SequenceMatrix (version 1.8). Pairwise-distances between the strains were calculated on the concatenated alignment in MEGA7 as p-distances with gaps treated as pairwise deletions. A haplotype network was inferred from the ITS1-5.8S-ITS2 marker using the pegas package (version 0.14) in R.

The high throughput sequence data is available in the NCBI SRA BioProject database under accession PRJNA733136 and the Sanger sequences are available in the NCBI GenBank under accession MZ310729-MZ310794.

### Cell culture characteristics

Selected phenotypical and culture characteristics of the *C. closterium* isolates were measured to assess whether they were related to the bacterial community composition (see below). Algal cell densities and cell dimensions were obtained for each culture using an Amnis ImageStream X^®^ Mark II (Millipore, MA-USA) flow cytometer. The LED (brightfield) was set to an intensity of 33.32 mW and the lasers of 642 nm (to detect autofluorescence of the cells) and 785 nm were set to 10 mW and 0.5 mW, respectively. Objects in the samples were acquired until at least a thousand diatom-sized objects were measured. The flow cytometer data were then analysed in the Amnis IDEAS 6.2.187.0 software (Millipore). Diatom cells were gated relying on the autofluorescence signal and brightfield aspect ratio. Gates were manually checked and adjusted for every sample using the images required for every object. Apart from diatom cell density, average diatom cell length and cell perimeter in the cultures were calculated using the skeleton mask (to correct for the curvature of the cells).

### Data analyses

Sequences matching chloroplasts (41% of the reads) and mitochondria (19% of the reads) were removed prior to analysis of the bacterial community data. Since blanks had up to four reads of an OTU, read counts below four could not be distinguished from noise/cross-contamination and were set to zero. Environmental samples (from in situ sediment or water) were rarefied to 2484 reads (the smallest number of reads in an environmental sample; Supplementary Fig. 1A). Samples from diatom cultures with <500 reads were discarded and the remaining culture samples were rarefied to 538 reads (the smallest number of reads in a culture sample; Supplementary Fig. 1B). The flattening of the rarefaction curves suggested that sufficient sequencing depth was achieved, in particular for the microbiomes of the diatom isolates. These datasets were used for all further analyses.

The rarefied reads of the diatom culture samples were analysed using Constrained Correspondence Analyses (CCAs). Separate CCAs on the bacterial OTUs were constrained by sets of spatial, phylogenetic, cell culture and environmental variables respectively. The spatial variables were constructed using the geographic locations from which the samples were taken. Distances between sample locations were spectrally decomposed into Principal Coordinates of Neighbour Matrices variables (PCNMs; vegan 2.4–4; [53]), orthogonal variables that represent the spatial patterns across different scales and can directly be used in the CCA. A similar approach was used to construct phylogenetically representative variables, with the genetic p-distances between the *C. closterium* strains being decomposed into PCNM variables. The nutrient concentrations, part of the environmental set, were log-transformed prior to the analyses. For each set of variables, the significant variables were then selected using the stepwise forward selection procedure in CCA, with the *p* value thresholds to include and exclude each variable set to 0.05 and 0.1, respectively. Variables with a variance inflation factor > 10 were removed. CCA-based variation partitioning was done with the thus selected variables (Supplementary Table 2) as explanatory variables in order to quantify the unique contributions of the host (cell culture and phylogenetic variables) and the environment (spatial and environmental variables) in explaining microbiome community structure. Permutation tests were used to assess the significance of the unique contributions of the host and the environment.

In addition, a CCA was performed on the environmental and culture samples together (both rarefied to 538 and converted to presence–absence). By constraining this CCA by sample type (environmental vs culture) whilst conditioning for the different locations, we could quantify the effect of the cultivation step on the microbiome communities (i.e. combined effect of single cell isolation, common garden environment and the diatom host). A Non-metric Multidimensional Scaling (NMDS) of the joint environmental and culture samples was constructed in two dimensions using Kruskal’s NMDS algorithm (isoMDS from the MASS package -version 7.3).

Functional annotation of the OTUs was done using FAPROTAX [54]. The taxonomic assignments of the OTUs obtained from MOTHUR (Version 1.32.1; [54, 55]) using the May 2013 GreenGenes training set [56, 57] were hereby mapped to metabolic and other ecologically relevant functions (e.g. denitrification or fermentation), based on the literature on cultured representatives. The functions were curated to remove overlaps between nested functions (e.g. ‘sulphate respiration’ overlaps with ‘respiration of sulfur compounds’) that would otherwise result in an overestimation of the functional diversity.

Randomisations of the microbiome community compositions were done using two different algorithms (Supplementary information: Illustration of randomisation procedures). First, a conservative randomisation procedure (‘swap procedure’, permatswap with the quasiswap method from vegan 2.4–4) was applied on the 16 S rRNA gene data of the diatom culture microbiomes, preserving both the matrix fill (the number of zero occurrences) and the column and row abundance totals. OTUs with an overall higher relative abundance in the dataset (i.e. higher column total) thus remained abundant and every microbiome always consisted of 538 reads (row totals). This procedure was constrained by sample station, i.e. this randomisation procedure was restricted to the microbiome data of isolates originating from the same station, which allowed to address the unique effect of host (cell culture and phylogenetic features) on microbiome structure. The randomisation procedure thus nullifies differences between hosts as well as selective effects due to bacterial interactions. Secondly, the identity of the bacteria was randomised to mimic a community assembly process independent of bacterial identity (‘lottery procedure’). To this end, the OTUs present in each observed diatom culture microbiome were replaced by OTUs present in the bacterial source community from which the diatom strain was isolated, with the probability that an OTU was selected from the source community being proportional to its abundance in this community (lottery dynamic). In addition, only OTUs which were observed at least once in one of the *Cylindrotheca* cultures were allowed to be selected to reflect that not all bacteria present in the source community can survive under the common garden conditions. OTUs were sampled without replacement (an OTU could only be selected once from the bacterial source community). The syntax for the randomisation procedures is provided on GitHub: https://github.com/willem-stock/microbiome_randomisations.git.

The randomisation procedures resulted in a thousand OTU datasets for each procedure, which were then structurally compared to the observed 16 S rRNA gene datasets by means of α- and β-diversity indices. The Shannon diversity index (*H*) was calculated for every community in all (observed and randomised) datasets for both taxonomic (OTU composition) and functional diversity (FAPROTAX-based function composition). The richness of the functional annotations was also calculated as the number of functions present in each community. The phylogenetic diversity within every community was calculated as mean pairwise phylogenetic distance (mpd function from the picante package) between all OTUs in a community, not taking the relative abundances of the OTUs in consideration. The differences between communities within the observed and randomised datasets were compared by means of β-diversity and other indices. The Bray–Curtis Dissimilarity index (vegdist function from the vegan package) between all community pairs based on the OTU incidences (presence/absence) was calculated in addition to the checkerboard score (C score; C.score function from the bipartite package). The C score [58] expresses the strength of co-occurrence patterns between OTUs in the different microbiomes within each dataset (exclusion increases the C score and reoccurring coexistence decreases it). The Bray–Curtis Dissimilarity index between communities was also calculated for the incidence of the functional annotations. The phylogenetic differences between communities were quantified as the mean pairwise phylogenetic distance separating the OTUs in two communities (comdist function from the picante package) without weighting by OTU abundances.

The neutral community model developed by Sloan et al. [59] was fitted to the observed community data and the randomised communities. In short, this model assumes that, in the absence of strong deterministic processes, the frequency at which bacteria are observed in a community will be proportional to their relative abundance in the source community. Hence, the frequencies at which OTUs were observed in the *Cylindrotheca*-associated communities were fitted to their relative abundances in the bacterial community from the location the diatoms were isolated from. As this analysis relies on frequencies of occurrence, it was limited to the three stations (ZW2, OS4, CA1) from which most diatoms had been isolated. The neutral model was fitted for each community dataset as in Burns et al. [60]. Goodness-of-fit for each dataset to the neutral model was evaluated by means of Efron’s Pseudo R-Squared.

## Results

Benthic *C. closterium* cells were isolated from sediment samples that were composed of silt to fine sand (median grain size 68.92 ± 96.75(SD) µm) with 2.41 ± 4.53% TOM. The interstitial water was marine to brackish (29.61 ± 6.61 PSU). The planktonic diatoms were isolated from water samples with a similar salinity (29.14 ± 2.49 PSU). Phosphate, ammonia and silicate were present at substantially higher concentrations in the interstitial water of the sediment samples than in the water samples (1985.58 ± 2017.73 compared to 149.65 ± 59.23 µg P-PO_4_^3−^; 5752.39 ± 3989.85 compared to 126.31 ± 121.65 µg N-NH_3_^+^; 4667.44 ± 2574.74 compared to 1743.07 ± 1192.64 µg Si-SiO_4_^4−^ respectively). The nitrite-nitrate concentrations were comparable between sample types (38.42 ± 69.55 compared to 20.04 ± 21.52 µg N-NO_2_^−^; 129.24 ± 228.22 compared to 116.50 ± 184.33 µg N-NO_3_^−^ respectively).

There were no clear relations between the genetic (haplotype) diversity of the *C. closterium* isolates and geographic location (Fig. 2).

The bacterial communities of the diatom cultures and the environmental samples which they were initiated from were characterised using high-throughput 16 S rRNA gene amplicon sequencing. The species richness of the microbiomes associated with the diatom isolates ranged from 1 to 19 OTUs within a single community and was on average thirty times lower than that of the environmental samples (expected number of OTUs after rarefaction of 8.03 ± 4.05 (SD) within a diatom associated community compared to 245.79 ± 106.39 within a source community). Overall, 130 different OTUs were observed across all the diatom associated bacterial communities and 1313 OTUs across all the different environmental samples.

**Fig. 3:**
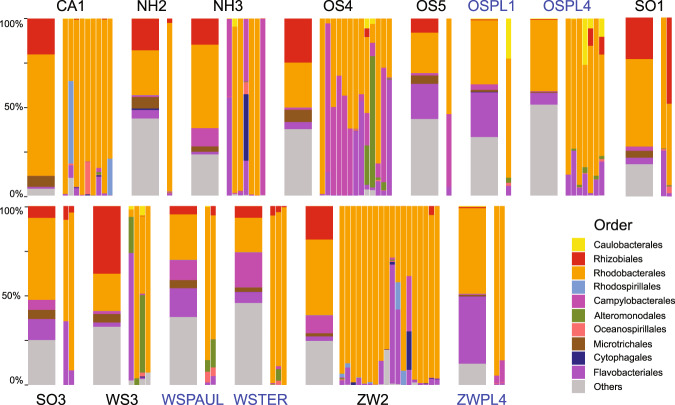
Composition of the bacterial communities in the source samples and in the *C. closterium* cultures. The relative abundances of bacterial orders (% of reads) for each source sample (wide bars) and the *C. closterium* cultures isolated from those samples (narrow bars) are shown. The top ten most abundant orders across the diatom cultures are indicated by different colours. The identifiers refer to the location where the source sample was taken (see supplementary table 1), with the water samples indicated in blue.

The bacterial communities in the *C. closterium* cultures were generally dominated by Rhodobacterales (Alphaproteobacteria), Campylobacterales (Epsilonproteobacteria) and Flavobacteriales (Bacteroidetes) (Fig. 3). In fact, all *C. closterium* microbiomes contained Proteobacteria, with *Yoonia-Loktanella* (in 82% of the *C. closterium* cultures), *Sulfitobacter* (in 36% of the *C. closterium* cultures) and *Marivita* (in 33% of the *C. closterium* cultures) being the most frequently observed. Flavobacteriales were observed in 85% of the *C. closterium* cultures, with *Winogradskyella* being the most prevalent genus (34% of the *C. closterium* cultures). The environmental samples exhibited a higher bacterial diversity than diatom culture samples (cf. above), but were also dominated by the same orders (Fig. 3).

**Fig. 4:**
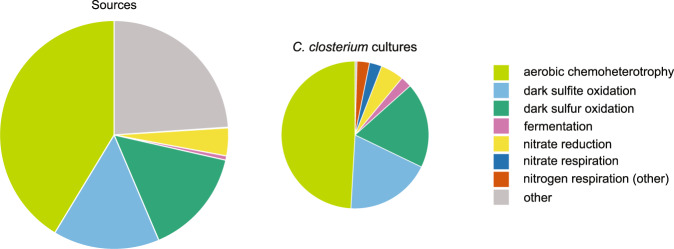
The selective enrichment of functions in the *C. closterium* cultures. The average relative abundance of the functions as annotated with FAPROTAX for the source samples (left) and the cultures (right) is shown. Only the top seven most prominent functions in the cultures are indicated by different colours.

Approximately half (47%) of the OTUs were assigned one or multiple functions using FAPROTAX. The bacteria in the *C. closterium* cultures were functionally distinct from those in the environmental samples. Of the 33 functions present in the environmental samples, 17 were found in the isolate samples: chemoheterotrophy, oxidation of sulfur compounds (sulfite and sulfur), fermentation and nitrogen (nitrate) respiration, being the most prominent ones (Fig. 4). Chemoheterotrophy was found in all but one of the diatom associated bacterial communities. Functions present in the sediment samples but not in the cultures included: degradation of complex carbon compounds such as lignins and aromatic hydrocarbons and the respiration of sulfur compounds. On average, 3.13 ± 1.41 (SD) functions were annotated per diatom associated bacterial community. The impact of both the environment (environmental conditions and geographic location) and host (phylogenetic identity and cell perimeter) on the structure of the *Cylindrotheca* isolate microbiomes was compared using variation partitioning (Supplementary Table 2). Host phylogeny and size (cell perimeter) had a smaller effect on bacterial community structure than environment. The unique contribution of the host was significant (8.2% of total variance explained; adj. *R*_´rel. ab_² = 3.1%; *p* = 0.003), with phylogeny in itself (retained phylogenetic PCNMs 1, 4 and 12 out of the 23; 6.7% of total variance explained) also being significant (*p* = 0.005) but not host size (mean cell perimeter, 1.6% of total variance explained; *p* = 0.203). The variation in the microbiomes uniquely related to the environmental characteristics was significant (19.9% of total variance explained; adj. *R*_´rel. ab_² = 11.1%; *p* ≤ 0.001). The unique contributions of geographic location (retained spatial PCNMs 1–4, out of the 7; 9.7% of total variance explained) and the environmental conditions at this location (retained variables: planktonic/benthic habitat, salinity and nitrite concentration; 8.3% of total variance explained) were individually also significant (both *p* values ≤ 0.001). The planktonic/benthic habitat distinction was the most important variable (see also Supplementary Fig. 2), explaining 6.7% of the variation by itself, but covarying strongly with the other variables. In the analysis including the microbiome data of both the environmental and culture samples, 3.6% (*p* = 0.001) of the variation in the bacterial communities could be attributed to sample type (environment or culture). The importance of sample type was similar when only presence/absence of the bacteria was considered (3.8%; *p* = 0.001).

**Fig. 5:**
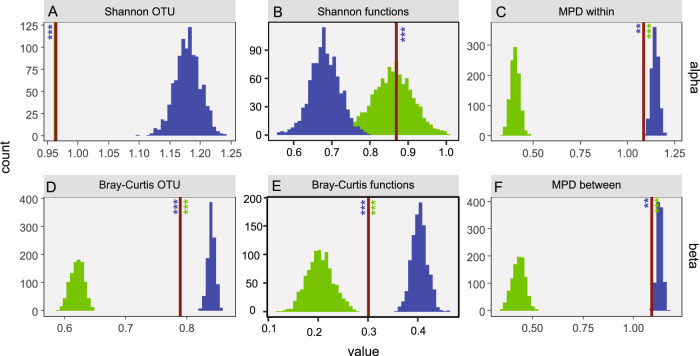
Pronounced structural differences between the observed and randomised communities. The histograms show the distribution of the mean indices for a 1000 lottery assembled communities (green) and a 1000 swap-based randomised datasets (blue). The red line shows the average value for the observed communities. The number of occasions that the values of the randomised data were more extreme to a single side of the observed data are indicated by asterisks (***all more extreme; ** between 1 and 10 values equal to or on the opposite side of the value obtained from the observed data). Alpha-diversity (within communities) indices are shown in (**A**–**C**) and beta-diversity (between communities) indices (**D**–**E**). From left-to-right then top-to-bottom: (**A**) Shannon diversity based on the OTU abundance data, (**B**) Shannon diversity based on the abundance of the annotated functions, (**C**) mean pairwise phylogenetic distance (MPD) within communities, (**D**) Bray–Curtis Dissimilarity based on the OTU incidence data, (**E**) Bray–Curtis Dissimilarity based on the presence/absence of the annotated functions, (**F**) mean pairwise phylogenetic distance between communities.

The observed bacterial communities associated with the *C. closterium* cultures were compared to randomised communities. The randomised communities were created [1] by randomising between diatom microbiomes originating from the same station and [2] by reassembling communities from the local bacterial source community. Both randomisations thus retained differences between stations but eliminated differences between hosts. While the first randomisation procedure eliminated selectional differences between hosts and interactions between bacteria, the second randomisation procedure eliminated selective effects altogether. The observed bacterial communities were markedly different from the randomised ones (Fig. 5). The observed OTU-based Shannon diversity (Fig. 5A) was lower than in all swap-based randomisations (*p* ≤ 0.001). Due to the nature of the lottery-based randomisations, Shannon diversity remained unchanged in those communities compared to the observed communities. As the mean number of OTUs per community remained the same in these randomised and the observed datasets (8.03 OTUs/community), the Shannon index is equivalent to Pielou’s evenness index [61] and the higher index in the randomised communities is strictly due to higher evenness. The mean Shannon index based on functional diversity was similar in the lottery-based and observed datasets (*p* > 0.05) with also the mean number of functions being similar (3.41 compared to 3.13 in the observed dataset; *p* ≤ 0.001). In contrast, the swap-based communities consistently had a lower functional diversity and less functions (2.81; *p* ≤ 0.001). The phylogenetic diversity within communities (mean pairwise phylogenetic distance) was markedly lower (*p* ≤ 0.001) in the lottery-based datasets yet slightly higher in the swap-based datasets (*p* = 0.002) compared to the observed communities. The phylogenetic diversity does not take relative abundances into consideration and as such is independent of the differential OTU abundances.

When considering differences in bacterial incidence (presence–absence) between communities, similar patterns were observed when comparing OTUs, functions and phylogenetic diversity (Fig. 5D–F). In all cases, the differences between the observed communities were smaller than in the swap-based datasets (*p* ≤ 0.001), but higher than in the lottery-based datasets (*p* ≤ 0.001). The C score, expressing the extent of species cooccurrence patterns [58], mirrored the results based on the OTU-based Bray–Curtis dissimilarity index: the lottery-based datasets had a consistently lower C score (0.644 ± 0.018; *p* ≤ 0.001), indicative for a more aggregated co-occurrence of OTUs in these datasets. All swap-based datasets on the other hand had a higher C score (0.893 ± 0.002; *p* ≤ 0.001) than the observed data (0.878). The lower function-based Bray–Curtis dissimilarity (*p* ≤ 0.001) and mean pairwise phylogenetic distance (*p* = 0.002) in the observed communities compared to the swap-based datasets illustrate a conservatism of functions and phylogenetic structure across communities. At the same time, communities were functionally (*p* ≤ 0.001) and phylogenetically (*p* ≤ 0.001) more distinct from one another than those simulated using the lottery-based approach. Comparing the OTUs, functions and phylogenetic diversity between observed and randomised communities on a per station basis resulted in the same overall patterns (data not shown).

**Fig. 6:**
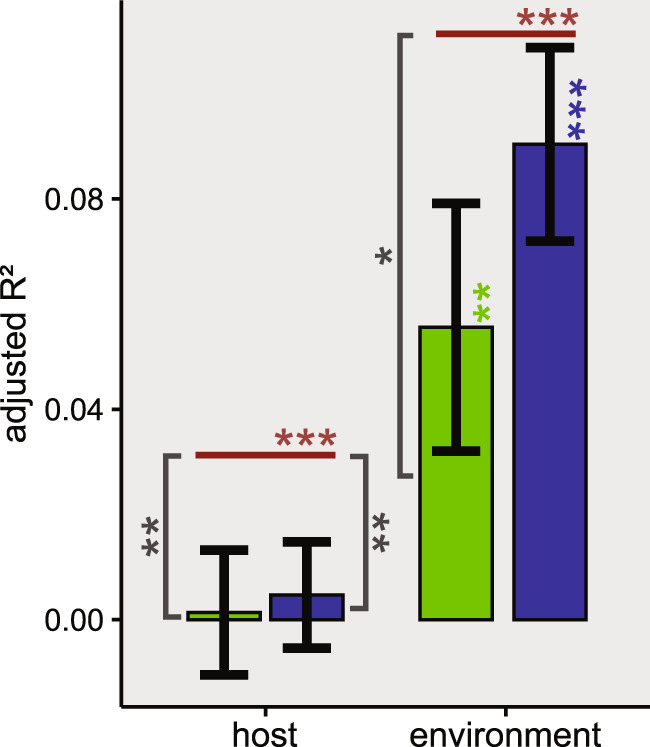
The amount of variation uniquely attributed to either the host or the environment in the observed and randomised datasets. The host and environment each uniquely explained a significant portion of the variation in the observed data (red lines, significance indicated by red asterisks). The average contribution of the host in the lottery-based (green bars) and swap-based (blue bars) randomisations was negligible and substantially smaller (grey asterisks) than in the observed dataset. Environment on the other hand remained an important factor in explaining the variation in the randomised communities (blue and green asterisks indicate the median significance of the swap-based and lottery-based randomisations respectively). Error bars are used to indicate standard deviation between randomisations. Significant differences in adjusted *R*² are indicated with asterisks as follows: ***p ≤ 0.001; **0.001 < *p* ≤ 0.01; *0.01 < *p* < 0.05.

The variation in each randomised dataset was partitioned as was done for the observed dataset (Fig. 6). The fraction of variation uniquely related to the host in both randomised datasets was close to zero (average adj. *R*²_swap_ = 0.005 ± 0.01; average adj. *R*²_lottery_ = 0.001 ± 0.01) and significantly smaller than in the observed dataset (adj. *R*_obs_ = 0.03; *p*_obs-swap_ = 0.003; *p*_obs-lottery_ = 0.009). The fraction of variation uniquely related to the environment was higher in the swap-based datasets (average adj. *R*²_swap_ = 0.09 ± 0.02) than in the lottery-based datasets (average adj. *R*²_lottery_ = 0.06 ± 0.02), but not as high as in the observed dataset (adj. *R*²_obs_ = 0.11; *p*_obs-swap_ = 0.015; *p*_obs-lottery_ = 0.13). In the overarching variation partitioning with both diatom-associated or randomised communities and the environmental communities, the contribution of sample type (environmental vs culture) was much larger (*p* ≤ 0.001) in the lottery-based datasets (0.062 ± 0.001) than in the observed (0.038), while the opposite was true for the swap-based dataset (0.034 ± 0.0005; *p* ≤ 0.001).

The goodness-of-fit for each dataset to a neutral assembly model was evaluated. The frequency at which the OTUs occurred in the observed *Cylindrotheca*-associated communities fitted relatively well (pseudo R-squared = 0.44) to their abundances in the in situ environmental communities. None of the swap-based randomisations fitted as good to the neutral model (pseudo R-squared = −0.34 ± 0.14; *p*_obs-swap_ ≤ 0.001). The lottery-based randomisations on the other hand fitted the model generally better than the observed communities (pseudo R-squared = 0.59 ± 0.058; *p*_obs -lottery_ = 0.012).

## Discussion

During the isolation of the individual *C. closterium* cells using micropipetting, only a fraction of the environmental microbiome was transferred into culture vessels. As a result of the isolation procedure [40] used in this study, the bacteria that were co-isolated with the diatom were most likely those attached to or associated with the diatom. Since the resulting diversity in the *C. closterium* cultures was low, the number of bacteria attached in situ to *C. closterium* cells is likely to be low as well, which would be in line with the microscopic observations made on the pennate diatom *Pseudo*-*nitzschia* by Kaczmarska et al. [28].

The common garden environment in which the diatoms were reared provided an equal playing field for bacteria to establish, only differing due to potential differences between diatoms and the bacteria community initially present. We investigated to what degree selective and neutral assembly processes shape taxonomic and functional community assembly in microbiomes associated with closely related representatives from the *Cylindrotheca closterium* marine diatom complex. Substantial variability in microbiome structure was observed between diatom cultures which, while originating from non-axenic, clonal isolates from different marine habitats, were reared in a common garden environment.

Variation partitioning of observed communities revealed that host-related selection plays a small yet significant role in shaping the structure of the observed diatom microbiomes. However, despite the common garden setting, the imprint of the local environment of the isolates on microbiome structure was larger than that of the host itself. The observed positive relation between the frequency at which bacteria were associated with their hosts and their abundance in the bacterial source communities suggests that this impact predominantly results from a lottery-like colonisation of environmental bacteria onto the diatom [59]. Since no allochthonous bacteria could replace the bacteria in the diatom-associated communities during the common garden phase, the imprint of the source communities persisted in the common garden setting.

Host-specific bacterial communities have been observed for many marine organisms (e.g. fish and their skin microbiome, [62]; sponges and their microbiome [63], yet rarely between such closely related hosts [16, 64]. Variation in microbiome structure was related to the phylogenetic relatedness of the hosts, suggesting that the host traits affecting the microbiomes harbour at least some degree of phylogenetic signal [65]. It is not unlikely that such traits are linked to exudate production by the diatoms [21, 66, 67], whereby more closely related hosts produce more similar exudates [68] which would have more comparable effects on the bacterial communities.

The extent to which the environment shapes the assembly and structure of microalga-associated bacterial communities, and more specifically the relevance of lottery dynamics in the assembly process, has been contested [30, 69]. In contrast with these two studies, we adopted a different experimental approach, starting from non-axenic algal isolates. As a result, we are comparing microbiomes that were partly assembled in situ, and thus also observed the imprint of bacterial community dynamics occurring in open systems where continuous colonisation and replacement is possible. Our results suggest that these dynamics, at least to a certain degree, conform a lottery assembly model, and reveal a significant imprint of the environment on the microbiomes. Our observations are thus in line with Eigemann et al. [70] and Filho et al. [71], who found that bacterial precolonization is a strong determinant of bacteria–algae associations, and are also consistent with the high variability observed on diatom cells in in situ natural environments [24, 31].

Pronounced differences were observed between the randomised communities obtained with the lottery model approach (mimicking a fully neutral assembly process) and the experimental communities, but also between the latter and the swap-based randomised communities (which fully eliminate the biotic effect on microbiome assembly). The substantially lower than observed mean phylogenetic diversity in the lottery assembled communities suggests a significant degree of phylogenetic overdispersion in the observed microbiomes. Assuming that phylogenetic relatedness correlates with ecological similarity, this could be indicative of competitive exclusion in the diatom microbiomes [72]. Overlapping resource utilisation could for instance prevent more closely related bacteria from co-occurring in the diatom phycosphere [73]. This contrasts however with the consistently higher than observed phylogenetic diversity found in the swap-based randomisations, which contain no host-related signal. Host-related selection thus also appears to simultaneously slightly limit phylogenetic diversity within the microbiomes by selecting for a more limited set of closely related, ecologically similar bacteria which are adapted to the host microhabitat [72]. Such bacteria could be avoiding competitive exclusion through for instance coevolved interdependencies and cross-feeding [74]. This would also explain the lower Bray–Curtis dissimilarity and C scores in the observed communities compared to the swab-based randomisations.

Only a restricted subset of bacteria present in the source communities was found associated with *C. closterium*. This subset of bacteria was able to establish itself in the common garden environment and withstands competitive displacement, i.e. habitat filtering, sensu Li et al. [75]. These bacteria can potentially persist by occupying the different available metabolic niches in the host-associated communities [76]. This is in accordance with the strongly conserved number of functions in the diatom associated communities. A pronounced functional selection [19] also explains the higher than expected functional evenness in the observed communities, despite their lower taxonomic evenness in comparison to the swap-based randomisations. Functions included heterotrophy, nutrient recycling and several anaerobic processes. As the diatom associated bacteria are largely dependent on the carbon fixed and released by their algal host [77], it was no surprise that heterotrophy was one of the most commonly detected functions amongst bacterial communities. Furthermore, the most frequently observed genera, namely *Yoonia-Loktanella* and *Sulfitobacter*, are all members of the *Roseobacter* group which is recognised for its capability to utilise even the most complex algal exudates [78]. Also the role of the bacteria in nutrient remineralisation is well established [79]. In contrast, the prevalence of anaerobic respiration processes was unexpected. It is unlikely, but not impossible [80] that oxygen was completely depleted in the cultures during the night due to algal and bacterial respiration. On the other hand, the FAPROTAX functional assignments are indirectly inferred from the taxonomically related bacteria and anoxic conditions were not proven to actually occur in the cultures, therefore it is important to interpret with caution.

It is worth noting that most of the reported interactions between *C. closterium* and its bacteria had a negative to neutral effect on the diatom growth [6, 14, 81] whilst for other diatoms more beneficial interactions with bacteria have been reported [6, 30]. It is likely that the importance of the host on the assembly process differs between diatom species and that the findings of this study might therefore not be generalisable. Secondly, it is unclear how the resulting differences in the microbiome impact the functioning of *C. closterium*. The effects of bacteria on the growth of *C. closterium* are highly strain specific [14] and the outcome of the assembly process is therefore likely to impact the fitness of the diatom. It is possible that host-specificity can result in fitness benefits to the host as was previously reported by Jackrel et al. [69] for some microalgae.

In this study, we analysed and compared the relatively simple microbiomes associated with different strains of the *C. closterium* species complex. The assembly process of the diatom associated bacterial communities was predominantly driven by neutral processes and as a result, the major differences between these communities resulted from variation in the bacterial source communities. Host identity and competitive exclusion between bacteria seemed to amplify these differences whilst a consistent functional selection and positive biotic interactions likely resulted in a higher similarity between communities. As a result of the different selectional processes, the variation between the diatom associated communities was higher than expected under a lottery dynamics scenario but less than between randomised bacterial communities. Our results suggest that in spite of the variability between hosts and source communities, the functions of the bacterial community on which hosts can rely are well conserved, but this remains to be experimentally validated.

## Supplementary information

Supplementary information
