# Supplementary material for: Selection constrains lottery assembly in the microbiomes of closely related diatom species
Source: ISME Commun. 2022 Feb 1;2:11. doi: 10.1038/s43705-022-00091-x (PMC9723743; doi:10.1038/s43705-022-00091-x)
Supplement: Supplementary file 1 — Supplementary information [file 43705_2022_91_MOESM1_ESM.docx]

# Supplementary information:

## Supplementary Table 1

Table 1: Overview of the diatoms isolated for this study. The number of isolates is shown for each station. Additional information about the stations is provided including geographical information and the sampled material.

| Location | Station | Latitude | Longitude | Sample type | *N° Cylindrotheca closterium isolates* | Other species |
| --- | --- | --- | --- | --- | --- | --- |
| North Holland (NL) | NH2 | 52.93520 | 5.01815 | Sediment | 1 |  |
|  | NH3 | 52.89533 | 4.91042 | Sediment | 6 |  |
| Oosterschelde (NL) | OS4 | 51.53022 | 3.94448 | Sediment | 11 |  |
|  | OS5 | 51.52024 | 3.93640 | Sediment | 1 |  |
|  | OSPL1 | 51.54938 | 3.86865 | Water | 1 | 2 (*Nitzschia* spp.) |
|  | OSPL4 | 51.53022 | 3.94448 | Water | 6 |  |
| Bay of the Somme (FR) | SO1 | 50.21445 | 1.56773 | Sediment | 2 |  |
|  | SO3 | 50.21512 | 1.56710 | Sediment | 2 | 1 (*Navicula phyllepta*) |
| Westerschelde estuary (NL) | WS3 | 51.35053 | 3.72623 | Sediment | 4 |  |
|  | WSPAUL | 51.35260 | 3.73078 | Water | 2 |  |
|  | WSTER | 51.33710 | 3.86612 | Water | 4 |  |
| Zwin, Salt marsh creek (BE) | ZW2 | 51.36550 | 3.36428 | Sediment | 18 | 1 (*Navicula phyllepta*) |
|  | ZWPL4 | 51.36658 | 3.36553 | Water | 2 | 1 (*Navicula* sp.) |
| Bay of the Canche (FR) | CA1 | 50.53735 | 1.59335 | Sediment | 9 |  |
|  |  |  |  | total: | 69 | 5 |

## Supplementary Table 2

Supplementary Table 2: variables used and retained in the variation partition analyses

## Illustration of randomisation procedures:

permatswap with the quasiswap

Submatrix of the rarefied abundance data

OTU3

OTU2

OTU1

permatswap

$$\begin{matrix} sample 1 \\ sample 2 \\ sample 3 \end{matrix}\left[ \begin{matrix} 10 & 0 & 10 \\ 0 & 50 & 0 \\ 30 & 0 & 10 \end{matrix} \right]$$

$$\begin{matrix} sample 1 \\ sample 2 \\ sample 3 \end{matrix}\left[ \begin{matrix} 0 & 20 & 0 \\ 30 & 0 & 20 \\ 10 & 30 & 0 \end{matrix} \right]$$

lottery

OTU1

OTU2

OTU3

$$\begin{matrix} sample 1 \\ sample 2 \\ sample 3 \end{matrix}\left[ \begin{matrix} 10 & 0 & 10 \\ 0 & 50 & 0 \\ 30 & 0 & 10 \end{matrix} \right]$$

OTU1

OTU3

Not present in the diatom associated bacterial communities

OTU4

OTU1

OTU2

OTU3

$$sample 1\left[ \begin{matrix} 10 & 10 \end{matrix} \right]$$

$$source sample 1\left[ \begin{matrix} 10 & 5 & 5 10 \end{matrix} \right]$$

## Supplementary figure 1


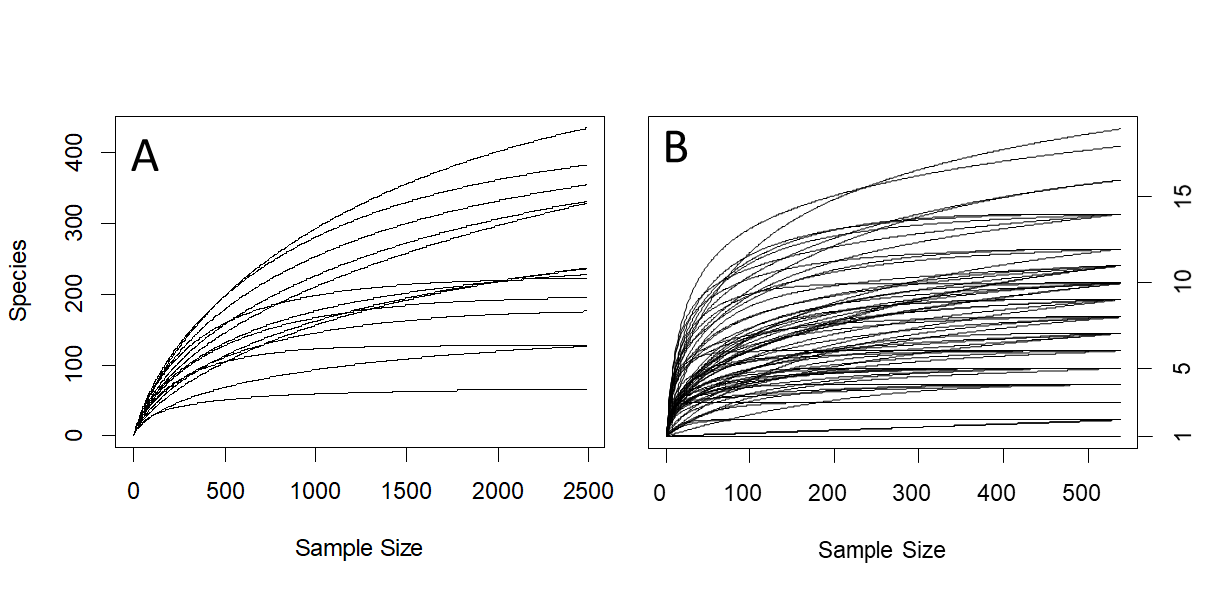


Supplementary figure 1: rarefaction curves. The OTU rarefaction curves of the environmental samples (A) and the diatom isolates (B), after removal of readcounts below four and to the depth (sample size) they were rarefied to for further analyses (see Material and Methods for more information).


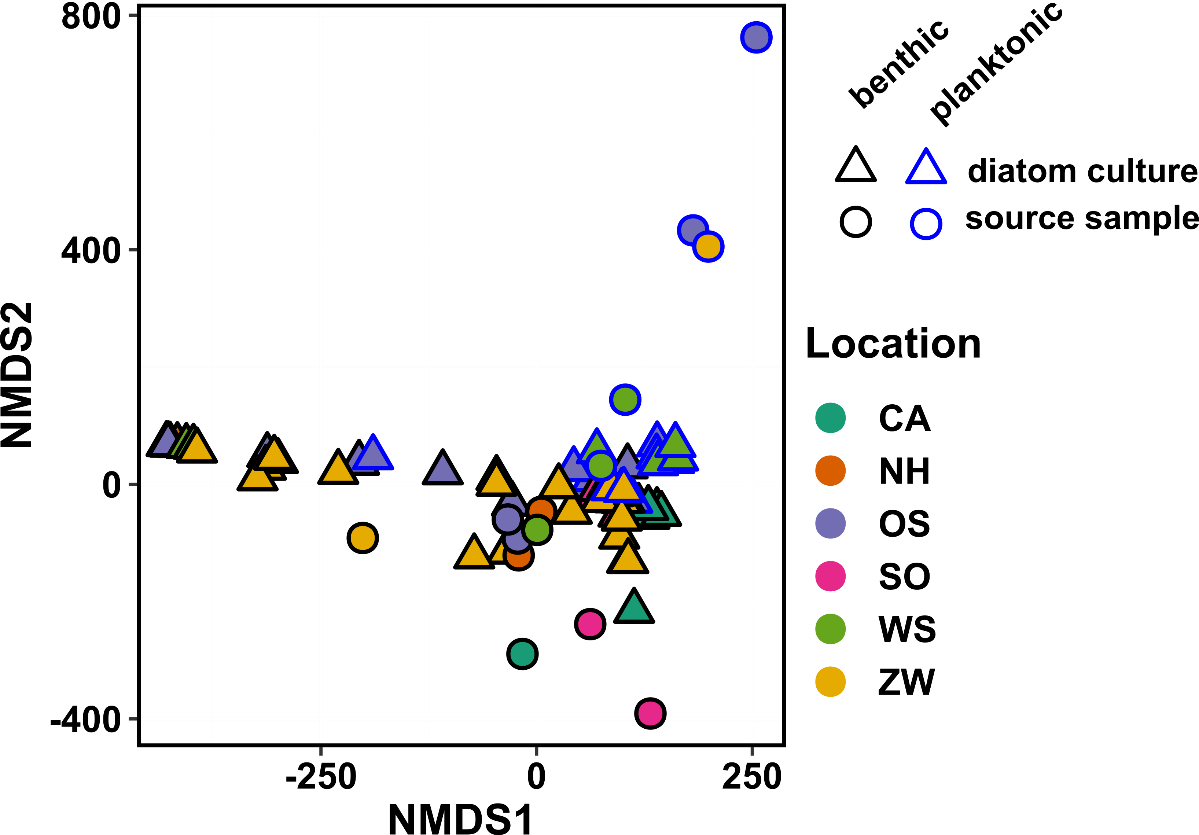
Supplementary figure 2: NMDS of the rarefied data sets of the bacterial communities from the diatom culture and the source samples. The ordination was constructed in two dimensions. The type of the sample (culture or source) is indicated by symbol shape, the original location of the samples by different fill colours, and the provenance of the source sample (benthic or planktonic) by the outline colour.
